# Efficient GAN-based Chest Radiographs (CXR) augmentation to diagnose coronavirus disease pneumonia

**DOI:** 10.7150/ijms.46684

**Published:** 2020-06-06

**Authors:** Saleh Albahli

**Affiliations:** Department of Information Technology, College of Computer, Qassim University, Buraydah, Saudi Arabia.

**Keywords:** Deep learning, Coronavirus, X-ray, Chest diseases, ResNet-152, Inception-V3

## Abstract

**Background**: As 2019 ends coronavirus disease start expanding all over the world. It is highly transmissible disease that can affect respiratory tract and can leads to organ failure. In 2020 it is declared by world health organization as “Public health emergency of international concerns”. The current situation of Covid-19 and chest related diseases have already gone through radical change with the advancements of image processing tools. There is no effective method which can accurately identify all chest related diseases and tackle the multiple class problems with reliable results.

**Method**: There are many potentially impactful applications of Deep Learning to fighting the Covid-19 from Chest X-Ray/CT Images, however, most are still in their early stages due to lack of data sharing as it continues to inhibit overall progress in a variety of medical research problems. Based on COVID-19 radiographical changes in CT images, this work aims to detect the possibility of COVID-19 in the patient. This work provides a significant contribution in terms of Gan based synthetic data and four different types of deep learning- based models which provided state of the art comparable results.

**Results**: A Deep Neural Network model provides a significant contribution in terms of detecting COVID-19 and provides effective analysis of chest related diseases with respect to age and gender. Our model achieves 89% accuracy in terms of Gan based synthetic data and four different types of deep learning- based models which provided state of the art comparable results.

**Conclusion**: If the gap in identifying of all viral pneumonias is not filled with effective automation of chest disease detection the healthcare industry may have to bear unfavorable circumstances.

## Introduction

In a recent study conducted by the World Health Organization (WHO), it was reported that 15% of the total death rates were due to respiratory diseases, it was also reported that about 450 million people (7% of the world's population) was affected by Pneumonia. The corona virus (Covid-19) which originated from Wuhan, China in December 2019 was declared a pandemic by the World Health Organization, it virus presented pneumonia like symptoms and high fever. Chest radiography is a method that has been used for a while in diagnosing abnormalities and irregularities concerned with the respiratory system 1. The images produce by these radiographs are only understood by specialist doctors in order to make the diagnosis, but with the outbreak of the Covid-19 came an increase demand for these doctors ranging from 2-3 doctors in small city hospitals to 20-30 in bigger hospitals managing the Covid-19 24/7. However, under developed countries don't mostly don't have access to these many doctors if any, an example is the continent of Africa in which most of the countries are under developed. The outbreak of Covid-19 made us realize that an intelligent system that could chest abnormalities effectively and efficiently was needed so these countries who didn't have access to the specialist doctors could still have a chance. This lead to researches made in order to find efficient ways radiographic images could be used to identify underlying respiratory diseases with high accuracy.

The Covid-19 and other respiratory diseases have seen a great progress due to image processing tools and researchers are using computer techniques and deep learning algorithms in order to achieve these breakthroughs 2,3,4. These algorithms were used to make radiographic image classification tools which are very useful in the detection of these respiratory abnormalities. If these tools are allowed to spread in the healthcare industry, they will bring a lot of benefits for the radiologists as it eases their work, clinic practitioners and even the patients who will use this technology in diagnosing and detecting these respiratory illnesses. The National Health Service reported that work load of radiologists has increased 30% over the last half decade, where as doctors of radiology only saw a 15% increase. Consequently, shortage in the radiologists and their amenities during the Covid-19 seems to continue if measured are not taken to moderate them. This has led to research and development of automated tools and machines which could detect these respiratory diseases in less time and with as little human effort as possible so as to lessen the burden on the radiologist who had to examine a lot of X-rays and due to human error could sometimes not be efficient especially since these radiologists are being over worked. These automated machines will read the X-rays and flag anyone with suspected abnormalities, which are the ones the doctors will focus on. This will not only eliminate the subjective opinion of doctors but also increase the rate at which the Covid-19 tests were done and also the amount done compare to when actual humans had to do them.

COVID-Net 2 is trained using COVIDx, a data set comprising nearly 6,000 X-ray images of 2,800 patients from a Kaggle challenge, as well as the COVID chest X-ray data set. COVIDx contains only 68 X-ray images from 19 confirmed COVID-19 cases. The data set also includes hundreds of non-COVID-19 viral infection images, like SARS, MERS, and influenza. COVID-Net also uses DarwinAI's explain-ability tools to highlight areas the model uses to justify its decision-making. COVID-Net was able to detect COVID-19 in 83.5% of cases. COVID-Net is designed to differentiate between COVID-19 and influenza, SARS, and MERS. Common onset symptoms of COVID-19 are fever, cough, myalgia, fatigue and dyspnea. In another case study, where they tried to differentiate COVID-19 from viral pneumonia: Influenza-A using CNN, overall accuracy of deep learning models was 86.7% for three groups: COVID-19, Influenza-A viral pneumonia and healthy cases from the perspective of CT Images.

In order to decrease the unnecessary waste of medical resources, it is important to accurately discriminate the bacterial pneumonia and viral pneumonia (COVID-19). In diagnosis of COVID-19, ground-glass opacity (GGO) in CT image is one of the most important factors to recognize the patients. Therefore, in another model it was expected that model can localize GGO in CT images, especially in the early stage patients and suspected patients. The diagnosis for a patient could be finished in 30 seconds, with parallel executions of thousands of tasks simultaneously. An online server is available for online diagnoses with CT images.

Findings indicate that DRNet learned to assess the correct features instead of learning image correlations. Moreover, the model provides reasonable clues on the factors for its judgements, which is of great help to assist doctors in diagnosis.^3^

This paper aims to effectively diagnose the chest diseases including Covid-19 and Pneumonia in the following classes, Atelectasis, Cardiomegaly, Effusion, Infiltration, Mass, Nodule, Pneumonia, Pneumothorax, Consolidation, Edema, Emphysema, Fibrosis, Pleural and Hernia. Most people who get COVID-19 have mild or moderate symptoms like coughing, a fever, and shortness of breath. But some who catch the new coronavirus get severe pneumonia in both lungs. COVID-19 pneumonia is a serious illness that can be deadly.

Thus, this work aims to provide the following contributions to the research community:

- Evaluate the performance of Chest Radiographs to analyze and diagnose of chest related disease including COVID-19;
- Multi-class classification models based on deep learning have been created to diagnose anomies on chest X-ray scans;
- A deep learning architecture and its hyperparameters have been optimized to improve the performance of the model;
- Insignificant features have been solved by adopting Generative Adversarial Network (GAN) based synthetic data;
- A comparative analysis with effective automation of chest disease detection is enhanced with respect to age and gender.

Rest of the paper is systematized in the following manner. In Section 2, the author has reported a dataset and its analytics. Section 3, elaborates the overall methodology of proposed solution and shows all deep learning models with different hyperparameters and compared the results. All 14 types of chest diseases are showed in the results and analysis Section 4. Finally, the conclusion of work is drawn in Section 5 with some discussion about limitations and future works.

## 2. Data and Evaluation

### 2.1 Chest X-ray Dataset

Deep learning tasks take the advantage of huge data and utilize computationally expensive training techniques to outperform the traditional machine learning tasks 4. These techniques need to have tremendous amounts of data in order to control latest advances. That said, if someone is trying to come up with the state-of-the-art applications, data needs to be concrete so superior that model can be leveraged. The COVID-19 virus causes fever, cough, fatigue and mild to severe respiratory complications, eventually leading to patient death. Each suspected case needs to be confirmed by the real-time polymerase chain reaction (RT-PCR) assay of the sputum. Although it is the gold standard for diagnosis, confirming COVID-19 patients using RT-PCR is time-consuming and has been reported to suffer from high false negative rates. On the other hand, because chest CT scans collected from COVID-19 patients frequently show bilateral patchy shadows or ground glass opacity (GGO) in the lung, it has been used as an important complementary indicator in COVID-19 screening due to high sensitivity. Due to fast progression of the disease, subsequent CT scans every 3-5 days are recommended to evaluate the therapeutic responses.

Our model used Chest Radiograph images (CXR) over CT scans for two reasons:

- Getting CXRs are more accessible for people than getting CT scans especially in rural and isolated areas. There will also be more potential data available;
- In the event radiologists and medical professionals become harmed from containing the virus for example: if they fall sick themselves, Artificial intelligence systems are vital to continue managing diagnosis.

A drawback of X-ray is that X-ray analysis requires a radiology expert and takes significant time which is precious when people are sick around the world. Therefore, developing an automated analysis system is required to save medical professionals valuable time.

For the purpose of analysis and model building the author utilized state of the art dataset provided by 5. This database contains total 108,948 X-ray images (frontal-view) of 32,717 unique patients. The author can divide the dataset into three classes Pneumonia (possibility of COVID-19), Normal or other chest related disease. The original dataset is classified in 9 major classes of chest related diseases as provided in the Table 1.

The rest of 84*,* 312 images belong to the normal patients having no disease. For the rest of the classes the author exploit the dataset from Kaggle challenge 2 which contains 503 Infiltration, 203 Effusion, 192 Atelectasis, 144 Nodule, 114 Pneumothorax, 99 Mass, 72 Consolidation, 65 Pleural Thickening, 50 Cardiomegaly, 142 Emphysema, 41 Edema, 38 Fibrosis, 14 Pneumonia and 5 images of Hernia. For COVID-19 the images collected was 337 from GitHub 6. Although the images of remaining classes are not enough for proper training, however the author has resolved the problem by exploiting synthetic dataset generated by state-of-the-art GAN model, the details can be seen in Section 3.

### 2.2. Technical Details of Training

**Figure 1:**
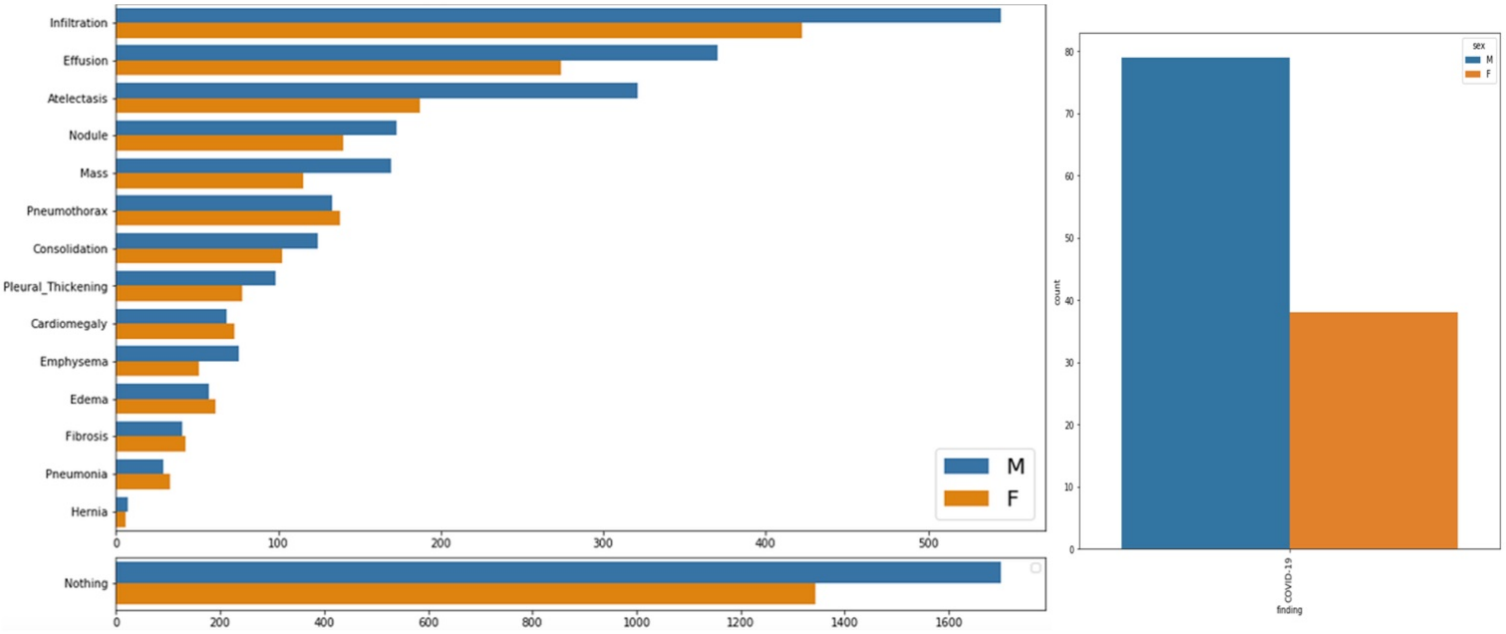
Gender wise distribution of chest related diseases.

In order to analyze the data, the author plotted it statistically using matplotlib from python. Here Figure 1 shows gender distribution for all the diseases other than COVID-19 which is shown in right side of Figure 1. It can be seen clearly that which gender is affected more by which disease i.e. Infiltration, Atelectasis, Consolidation, Emphysema, Hernia, Effusion, Nodule, Pleural Thickening and COVID-19 affect male more, than female. On the other hand, Pneumothorax, Edema and Emphysema affect more females than male.

**Figure 2:**
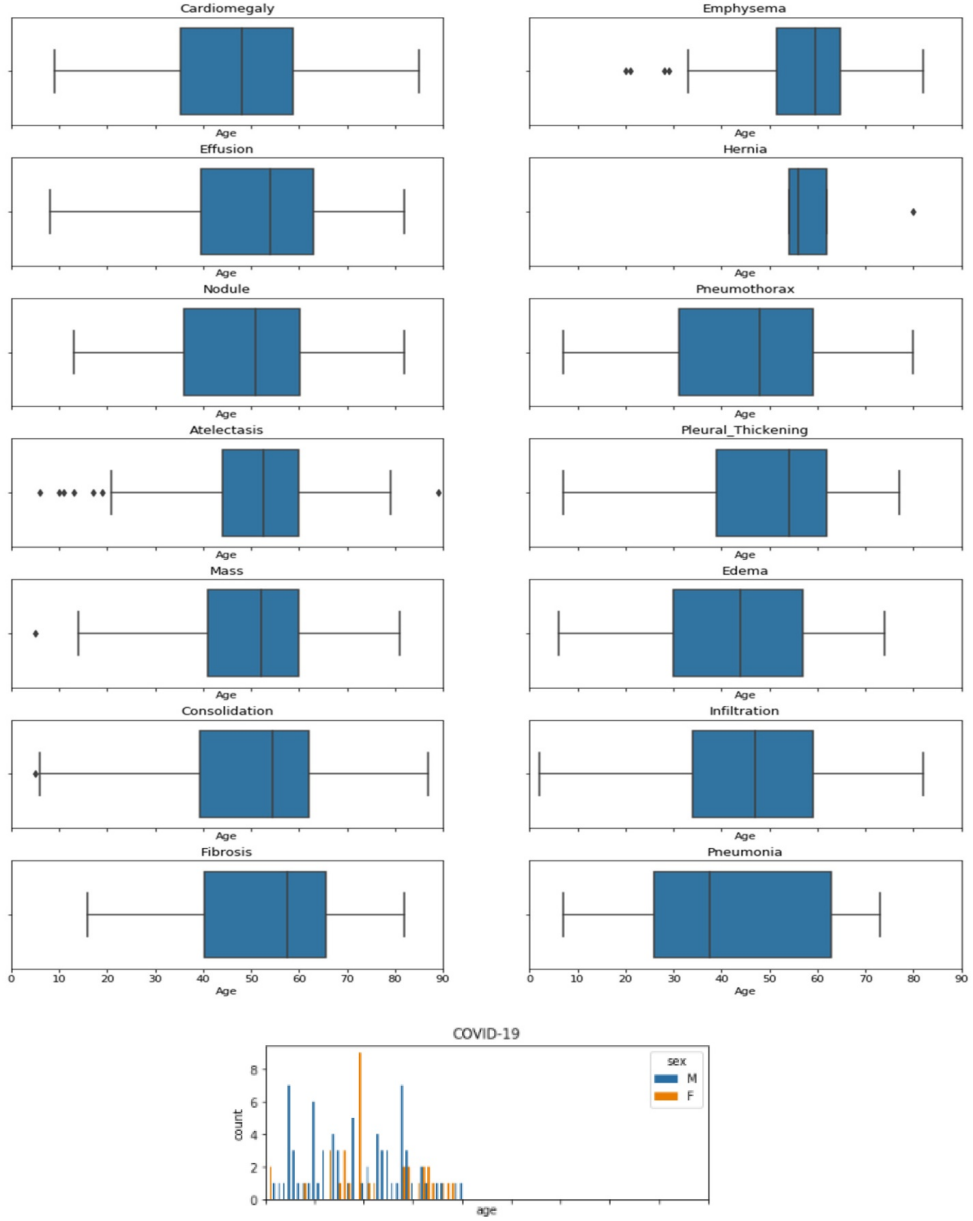
Age wise distribution of Covid-19 and boxplot chest related diseases.

Figure 2 shows number of patients of each gender with respect to age for all diseases. It can be seen that Cardiomegaly and Effusion shows same trends of effecting people older than 10 and has most cases between the age of 35 to 60, with medians at 50 and 55 respectively. Atelectasis affects people above the age of 15 and makes a bell shape distribution above this age with a constant distribution below age of 15. Mass extremely affects children, with a fair number of cases in teenagers and adults. Emphysema affects teenagers mostly and affects adults of age below 27-28. Here it can be seen that pneumonia affect only people under 12 while pneumonia caused by COVID-19 affect people of all ages. So, if a child under 12 has pneumonia there can also be the chances of COVID-19.

## 3. Proposed contribution

### 3.1 Data Preprocessing

It can be seen from the statistics provided in previous section that data is highly imbalances with 84,312 images of normal x-ray images. Imbalanced dataset can cause inaccurate and false predictions, so the author exploited the power of generative adversarial Networks (GANs) for generating our own dataset for classes having fewer images.

GAN model used for image generating purposes was proposed by Jianmin Bao et al. 7. Proposed GAN model is a combination of variational auto-encoder and GAN which generate fine grained images. The details of the network can be found in 7. Figure 6 shows a generic structure of proposed GAN model. In the figure x and x' are input and generated image. *E and G* are encoder and generator network, respectively. *z* is the latent vector. *c* is the condition, such as attribute or class label.

Images were generated for all classes with a smaller number of images even for COVID-19 images. After generating the dataset, it was necessary to validate our results from professional. In order to do so opinion of 5 expert doctors was utilized. Each doctor rated every image of specific disease from 0 to 5 (0 means highly irrelevant and 5 means highly accurate). Then score from all doctors for each image was averaged and images with score less than 4.5 were discarded.

As “no disease” and “infiltration” class has most images, in order to get equal samples of each class we took random samples from these classes and at the end we got all classes with same number of images each class which is 5000 i.e. dataset of 80,000.

For pre-processing, the author distributed images into training and validation folders where each folder contains sub-folder with class name 8.

These sub-folders were used for modeling. Before feeding data to the model all the images were reshaped into same dimensions of (150, 150, 3) and normalized. Augmentation was also applied in order to cover wide range of X-ray images that may come across the model in field. All classes were labeled using one-hot-encoding and the array was reshaped into (128, 128, 3). These arrays were saved into pickle files to be used later for modeling. As in the X-ray images, it is difficult to identify a specific pattern for each class. Here we wondered that “can deep learning identify these patterns to classify images correctly”.

### 3.2 Deep learning models

In order to get maximum accuracy, the author used different scenarios and parameter selection for these scenarios which was done on basis of previous models from literature which worked best. The accuracy measured and the results compared as follows:

Scenario 1: Deep Learning Model with Image Argumentation

**Figure 3:**
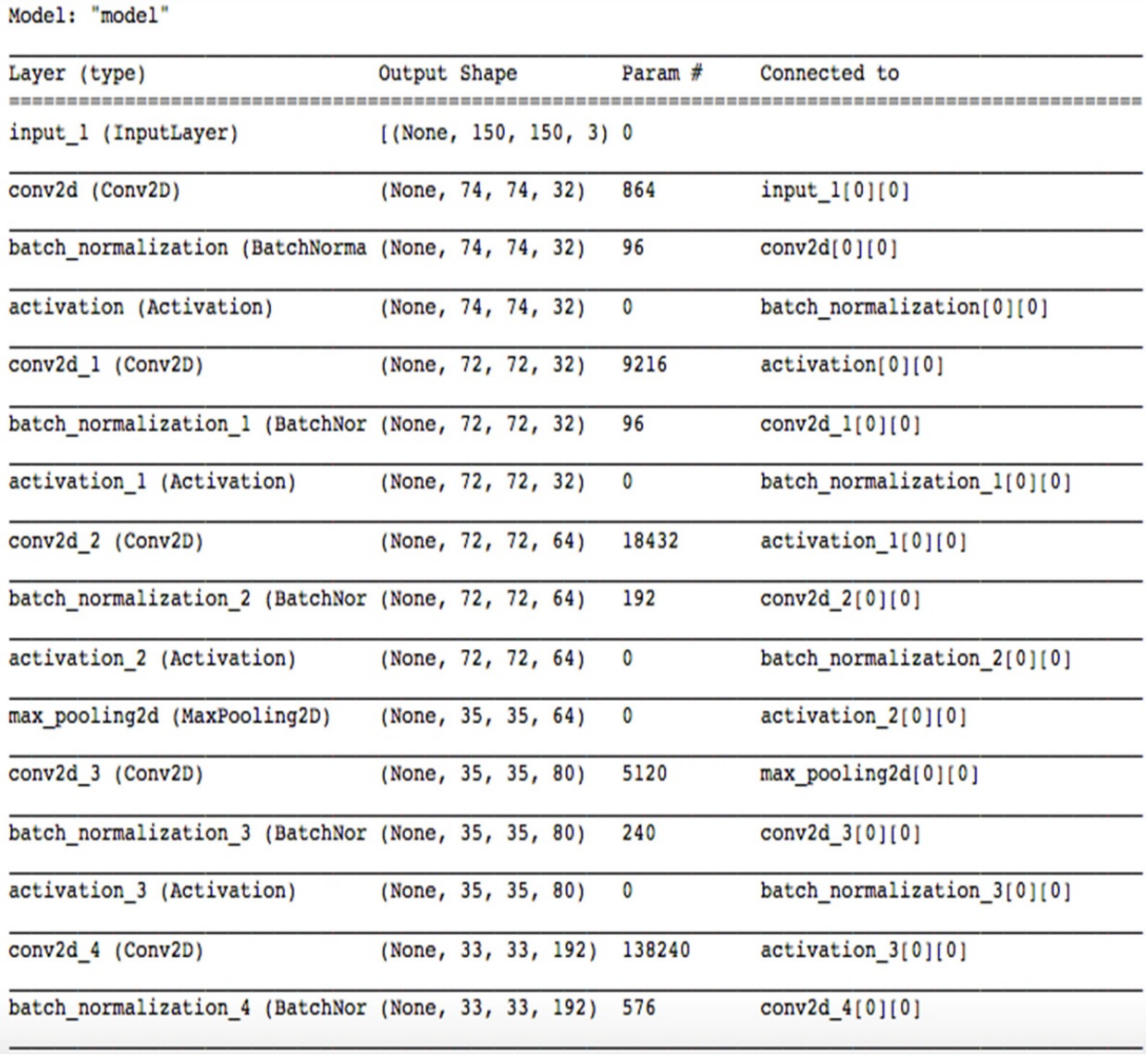
Architecture diagram of model-1.

The structure of Model 1 can be seen in Figure 3. Model contain 4 convolutional layers, where each layer is followed by max-pooling layer, at the end 1 flatten, 1 drop-out and 2 fully connected layers were added. For activation function Leaky-ReLU was used along with ADAM optimizer in order to make model capable to classify 15 classes. Input is images collected and generated through GANs and was fed to convolutional layer. While the output is 15 node layers for classifying 15 classes. Other model details can be seen in Figure 3, i.e. number of filters, parameter values and input/output. For Augmentation the following parameter ranges were used:

- rotation range = 40
- height / width shift range = 0.2
- zoom / shear range = 0.2
- fill mode = nearest
- horizontal flip = True

Scenario 2: Transfer Learning Using Inception-V3

**Figure 4:**
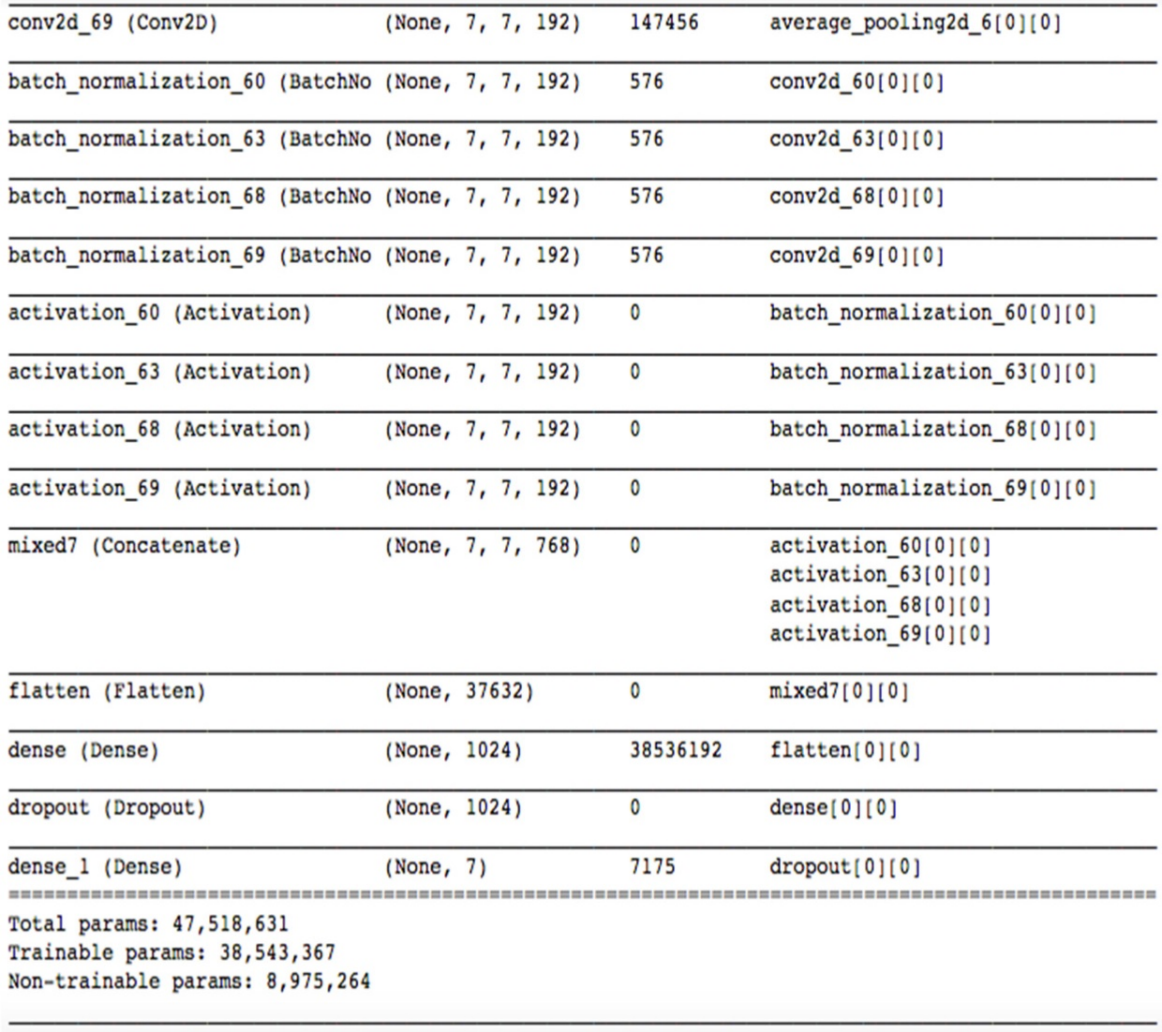
Architecture diagram of model-2.

A state-of-the-art pretrained model named inceptionV3 9 is used for transfer learning. Motivation behind using inceptionV3 as transfer learning as it gives lower error rate and was runner up in “ImageNet Large Scale Visual Recognition Competition 2015”. It contains 1024 fully connected layers with ReLU as activation function, except for the output layer where Softmax is used. Due to the dropout of 0.2, it prevents overfitting. InceptionV1 previously named GoogleNet was introduced by Szegedy et al. 10. After that many variants of this model came along with Inception V2 11 with an addition of batch normalization. After that idea of factorization was added by the same author and Inception-v3 was introduced. This is a powerful model which can solve heavy computations without introducing much complexity. Input and output for this model is same as described in Model 1. Figure 4 shows details of Model 2. Augmentation was done in same manner described in Model 1.

Scenario 3: ResNet model without image augmentation

For experimental purposes a deep convolutional model was derived from Res-Net-152 12. It consists of 152 hidden layers and ReLU as activation function. In order to enhance overall performance it has residual connections. In contrast, ReLu contains multiple residual connections, each of 2D. Each residual unit can be expressed as equation 1:


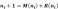
(1)

**Figure 5:**
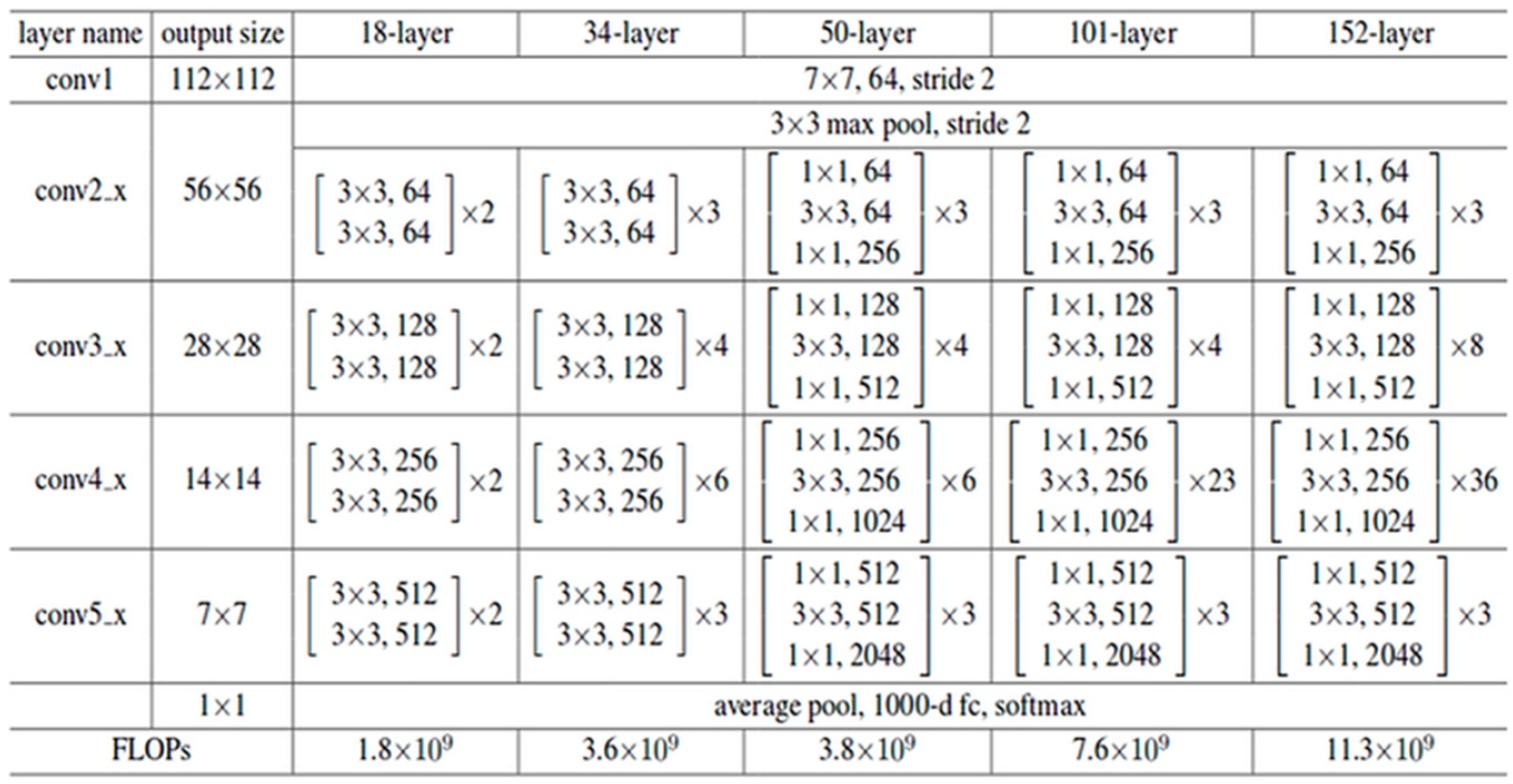
Architecture diagram of model-3.

**Figure 6:**
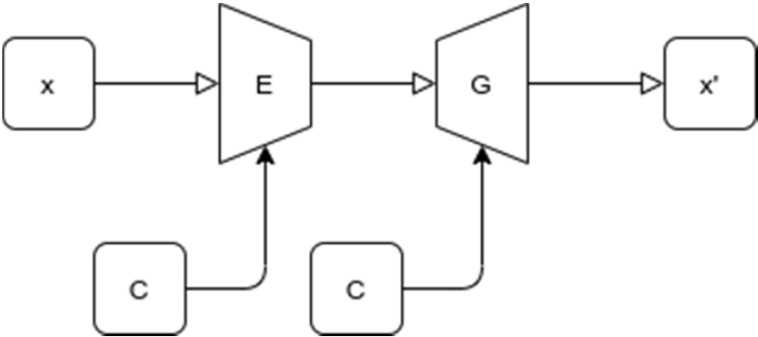
Generic structure of proposed GAN model.

Here 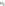
is input residual unit, while 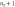 is output of t^th^ residual Unit. Here 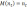 is identity mapping function and *R* is residual mapping non-linear function. Basic idea behind ResNet is that optimal value for R should be learned by model in accordance to inputs 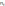
which are provided by shortest connection. SoftMax was used at output layer with 15 classes. Input and output for this model is same as described in Model 1. Model was built on Keras using TensorFlow. Remaining details are shown in Figure 5.

Scenario 4: Model-1 with 9 targeted classes

Architecture of Model 1 is used here but only with 9 basic classes from slandered dataset. Data augmentation is done on same manner as described in Model 1. Input for this model is same as used in Model 1 whereas output is 8 node layers to classify 9 classes.

## 4. Results and Discussion

**Figure 7:**
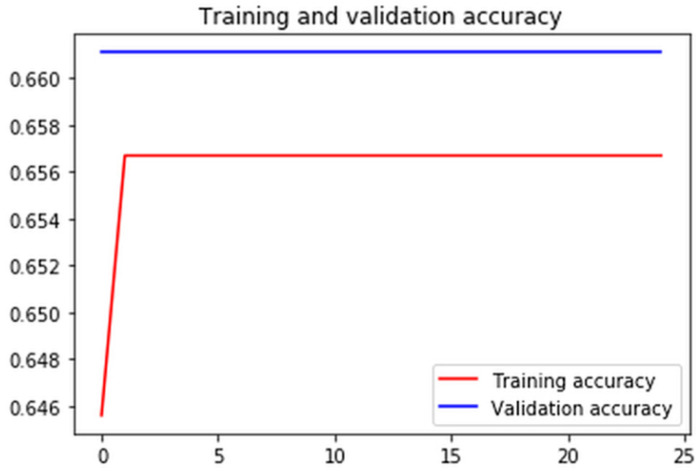
Training and validation accuracy of the convolutional neural network using image argumentation.

**Figure 8:**
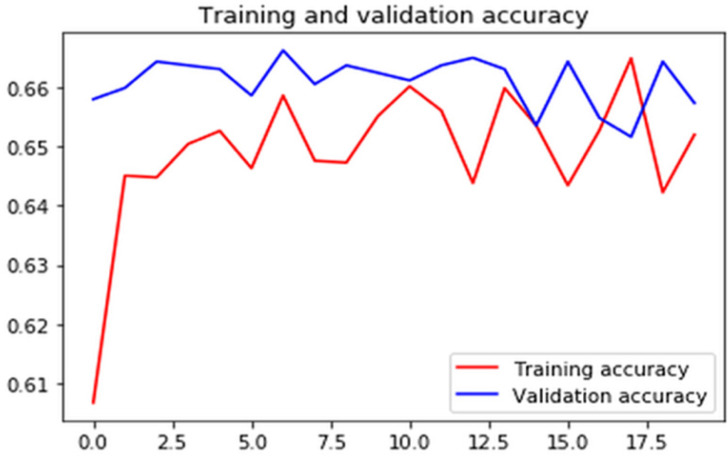
Training and validation accuracy of the transfer learning model using inceptionv3.

**Figure 9:**
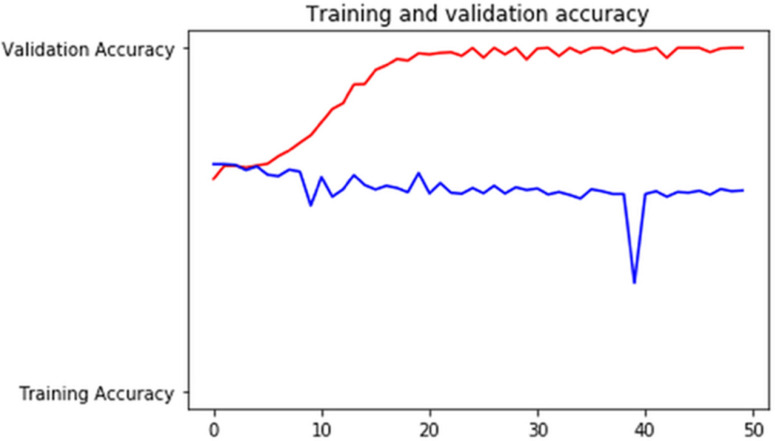
Training and validation accuracy of the ResNet-152 based deep learning model.

**Figure 10:**
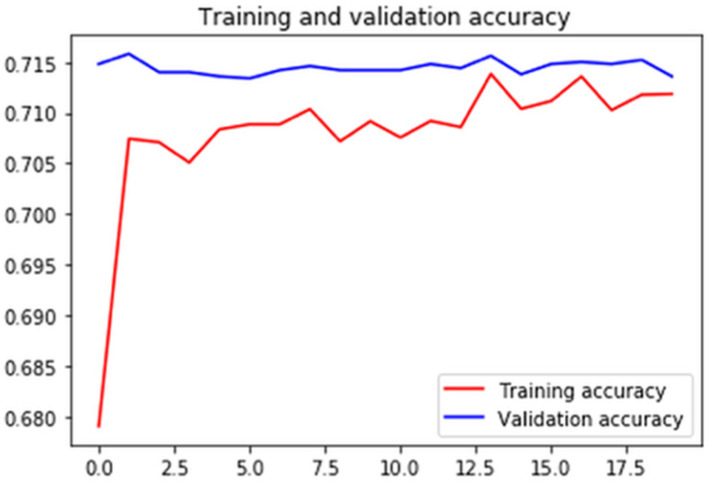
Training and validation accuracy of the deep learning model with 9 targeted classes.

Figures 7-10 show detailed analysis of training and validation accuracies for model 1, 2, 3 and 4 respectively. Model of Figure 7 was trained for only 100 epochs with variable learning rate and for such a smaller number of epochs training and validation accuracy remains almost constant at 83% and 80%. More epochs might increase accuracy of models. In Figure 8 it can be seen that at some point validation accuracy of 82% was attained. Thus, means transfer learning model can increase performance/accuracy by almost 2-3%.

Training and validation accuracy for RasNet came out to be 90% and 85-87% respectively and it can be seen in Figure 9. This is a huge improvement in accuracy. Training and validation accuracy for Model 4 is shown in Figure 10 and it can be seen that validation accuracy came out to be 80.12% which is not much of an improvement even when classes are reduced to 8 for Model 1. This also shows that our dataset normalization technique is state-of-the-art and provides significant results improvement for overall 15 classes.

### 4.1 Comparative Analysis

**Figure 11:**
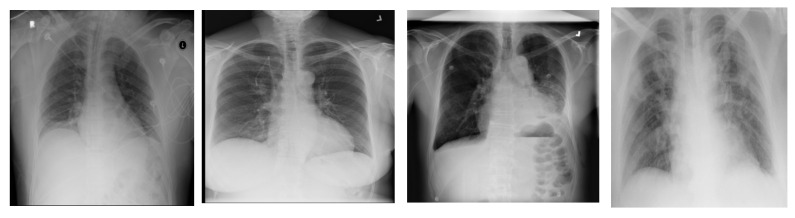
Heatmap of X-ray images with (from left-to-right) Edema, Effusion, Emphysema, COVID findings.

As we have already discussed in Section 1, that there is not much work present which targeted all 14 types of chest diseases plus COVID-19 caused pneumonia. Although, three papers performed such experiments one of them contributed in terms of data, second provided results with 15 classes but with highly normalized data and 3rd provided reasonable accuracy but still used normalized data. Table 2 provides comparative analysis of training and validation in terms of accuracy. It shows that the validation accuracy increases from around 66% to around 81%. However, with more training data with balanced classes, the model accuracy can increase significantly. The sample of heat-maps of GAN generated data can be visualized in the Figure 11.

## 5. Conclusion

Coronavirus Disease-2019 (COVID-19) has broadly spread everywhere throughout the world since the start of 2020. It is exceptionally infectious and may prompt intense respiratory misery or numerous organ disappointments in serious cases. This investigation plans to build up an automatic structure to identify Pneumonia (possibility of Covid-19) utilizing chest CT and assesses its performance. Detection of chest diseases from X-ray images is a changeling task and requires attention from research community and industry. There have been only few works which provided reasonable work to cater the problem under discussed. The dataset of x-ray has collected from a standard dataset and combined it with the few samples of rare classes from Kaggle challenge. The samples of rare classes are than generated by exploiting GAN based model. These data samples are than verified by an average scoring method with the help of expert doctors from the relevant filed. Afterwards, four different types of models were trained in order to conduct the experiments. The author concluded that data augmentation may increase the overall accuracy of model; however, in case of balanced data the ResNet based model provides 89% accuracy which is more than the previous state of the art models. The major limitation of this work is that the x-ray image is only from frontal view but according to some experts it is required to have the lateral view for accurate results. Our future work will be the steps towards the real time data collection of lateral view and rare classes. A detail exploration of deep learning approaches if need to identify coronavirus illness and recognize it from community acquired pneumonia and other non-pneumonic lung ailments utilizing chest CT.

## Figures and Tables

**Table 1:** Nine classes of chest related diseases in the dataset used

Class	Count	Class	Count
Atelectasis	5,789	Mass	6,046
Cardiomegaly	1,010	Nodule	1,971
Effusion	6,331	Pneumonia	1,062
Infiltration	10,317	Pneumothorax	2,793
Covid-19 = 337			

**Table 2:** Extreme values in CAD

Model	Training Accuracy	Validation Accuracy
Scenario 1: CNN with Image Argumentation 13	0.6567	0.6611
Scenario 2: Pre-trained Model, InceptionV3 with Image Argumentation 5	0.6551	0.6643
Scenario 3: ResNet model without image augmentation 14	0.89	0.5981
Scenario 4: Model 2 with reduce target to sample > 100	0.810	0.892
